# Targeted Neoadjuvant Therapies in HR+/HER2−Breast Cancers: Challenges for Improving pCR

**DOI:** 10.3390/cancers13030458

**Published:** 2021-01-26

**Authors:** Nandini Dey, Jennifer Aske, Pradip De

**Affiliations:** Translational Oncology Laboratory, Avera Cancer Institute, Sioux Falls, SD 57105, USA; nandini.dey@avera.org (N.D.); Jennifer.Aske@avera.org (J.A.)

**Keywords:** breast cancer, ER+, neoadjuvant, cell cycle, apoptosis

## Abstract

**Simple Summary:**

A high pathological complete response in the neoadjuvant setting is directly associated with a better overall response. A favorable prognosis is achieved when preoperative chemo or endocrine therapy succeeds in achieving a high pathological complete response (total eradication of tumors in the breast and the lymph nodes). Approximately 70% of breast cancers are ER-positive. The growth and progression of ER-positive breast cancers are critically dependent on estrogen receptor signaling. Although endocrine therapies (tamoxifen, an aromatase inhibitor, and fulvestrant) in ER-positive breast cancers are the backbone of adjuvant setting, the efficacy of such therapies in terms of achieving a pathological complete response is not encouraging in the neoadjuvant setting. Similar results are observed following targeted therapies in a neoadjuvant setting. Reviewing the literature in the context of different therapies of ER-positive breast cancers in the neoadjuvant setting, here we propose two hypothetical strategies to induce apoptosis based on the background of genomic alterations in the tumor tissues.

**Abstract:**

A strong association of pCR (pathological complete response) with disease-free survival or overall survival is clinically desirable. The association of pCR with disease-free survival or overall survival in ER+/HER2−breast cancers following neoadjuvant systemic therapy (NAT) or neoadjuvant endocrine therapy (NET) is relatively low as compared to the other two subtypes of breast cancers, namely triple-negative and HER2+ amplified. On the bright side, a neoadjuvant model offers a potential opportunity to explore the efficacy of novel therapies and the associated genomic alterations, thus providing a rare personalized insight into the tumor’s biology and the tumor cells’ response to the drug. Several decades of research have taught us that the disease’s biology is a critical factor determining the tumor cells’ response to any therapy and hence the final outcome of the disease. Here we propose two scenarios wherein apoptosis can be induced in ER+/HER2− breast cancers expressing wild type TP53 and RB genes following combinations of BCL2 inhibitor, MDM2 inhibitor, and cell-cycle inhibitor. The suggested combinations are contextual and based on the current understanding of the cell signaling in the ER+/HER2− breast cancers. The two combinations of drugs are (1) BCL2 inhibitor plus a cell-cycle inhibitor, which can prime the tumor cells for apoptosis, and (2) BCL2 inhibitor plus an MDM2 inhibitor.

## 1. Current Status of Systemic Neoadjuvant Therapy

The clinical management of estrogen receptor-positive breast cancer (BC) in the neoadjuvant setting has evolved with the advancement of the knowledge regarding the estrogen-receptor signaling pathway. The overarching burden of blocking estrogen-mediated signals in cancer cells has been achieved by three means. They are by [1] modulating the estrogen-receptor, [2] reducing the ligand-binding to the receptor, and [3] decreasing the number of estrogen-receptors. Earlier, tamoxifen was administered in patients to inhibit estrogen-receptor activation. In later years, synthesis blockers for estrogen, AI (aromatase inhibitor), and an estrogen-receptor degrader, fulvestrant, were approved in clinics.

Historically, neoadjuvant/presurgical therapies refer to the administration of treatments before surgery and have been used for the last couple of decades to downstage locally advanced/ unresectable primary breast cancers to make them operable [1,2]. However, several studies have highlighted the significant role of neoadjuvant endocrine therapy (NET) as an alternative option to chemotherapy in hormone receptor-positive (HR+)/human epidermal growth factor receptor 2-negative (HER2−) breast cancers (BC), especially in the postmenopausal setting [3,4,5]. Response to neoadjuvant endocrine therapy has been shown to correlate with the expression levels of ER, as quantified by the Allred score (according to CAP guidelines). Tumors that are more likely to respond to NET have high ER expression (Allred score of 7 or 8) and a low expression of Ki67 (<10, the proliferative index). Neoadjuvant systemic therapy aims to improve surgical outcomes by causing primary tumor shrinkage by providing effective chemo/hormonal therapies. Chemotherapy has been used traditionally as neoadjuvant therapy. Recent prospective neoadjuvant chemotherapy with docetaxel and cyclophosphamide showed that this chemotherapy regimen achieved a high clinical response rate in patients with stage II to III estrogen receptor-positive (ER+)/HER2− disease [6]. Future trials will examine the relative comparative efficacies of chemotherapy and endocrine therapy in ER+BC with low estrogen-receptor expression in the neoadjuvant setting.

With the advent of a more targeted therapy directed towards hormone receptors, the standard-of-care evolved to antiestrogen receptors. Clinical data showed the efficacy of different endocrine agents, including aromatase inhibitors (AIs), estrogen receptor modulators (tamoxifen), and estrogen receptor degraders (fulvestrant). The identification of optimal agents can result in stratified treatment for both premenopausal and postmenopausal women. Combining CDK4/6 inhibitor(s) with AI or fulvestrant yields promising effects for postmenopausal patients with advanced or metastatic BC [7,8]. Similar outcomes are being translated to neoadjuvant settings and offer possibilities to explore requirements of drug resistance mechanisms and new drug development [9]. In the era of precision medicine, a smart drug combination will evolve based on the functional relationship of signaling pathways and the genomic landscape of ER+BC. Typically neoadjuvant chemotherapy or neoadjuvant endocrine therapy followed by surgery and adjuvant endocrine therapy is the choice of therapeutic strategy. The choice of chemotherapy or endocrine therapy as neoadjuvant treatment is subject to disease characteristics and patient subtypes.

Data from trials in the neoadjuvant setting demonstrated that when compared with neoadjuvant chemotherapy, neoadjuvant AI has significantly lower toxicity. AI has comparable efficacy to neoadjuvant chemotherapy in terms of pCR (pathological complete response), ORR (objective response rate), and BCS (breast-conserving surgery), indicating the possibility of this well-tolerated strategy, especially for postmenopausal women [10]. The P024 trial demonstrated that letrozole (one of the AIs) was superior in terms of CRR (clinical response rate) and BCS over tamoxifen [10]. A meta-analysis of seven randomized trials illustrated that neoadjuvant AI treatment showed better efficacy than tamoxifen [4]. Neoadjuvant AI treatment also showed better efficacy than neoadjuvant chemotherapy and is associated with similar response rates to neoadjuvant combination chemotherapy with significantly lower toxicity [4,11].

The data from several trials have helped in the clinical comparison of the efficacies of different AIs, exemestane, letrozole, or anastrozole used in the neoadjuvant settings [12,13]. The stage II/III ACOSOG Z1031A trial in postmenopausal women with ER+ BC (Allred score, 6 to 8) showed a comparable response (neither superior nor inferior) with exemestane, letrozole, or anastrozole for 16 weeks. The three AIs had clinically and biologically equivalent effects, as the CRR was 60%, 72%, and 68%, and the expression of Ki67 in their tissue samples was 87.2%, 82.1%, and 78%, respectively [14]. It has also been reported that clinical efficacy did not significantly differ among the three AIs (letrozole vs. anastrozole vs. exemestane) in NET settings.

NCCN guidelines regarding estrogen receptor degrader mainly recommend fulvestrant as a first-line endocrine treatment for ER+ metastatic BC that progressed on either tamoxifen or AI. Reports have demonstrated the appropriate treatment dosing and clinical value of fulvestrant in NET. The phase 2 NEWEST trial showed that 500 mg fulvestrant had significantly greater early reduction in the levels of ER (−25.0% vs. −13.5%, *p* = 0.0002) and the expression of Ki67 (−78.8% vs. −47.4%, *p* < 0.0001) than 250 mg dose schedule [15]. The UNICANCER CARMINA 02 French trial (UCBG 0609; a randomized phase 2 neoadjuvant trial evaluating anastrozole and fulvestrant efficacy for postmenopausal, ER+/HER2− patients) clearly showed that both drugs are effective and well-tolerated as NET in postmenopausal women [16].

The clinical efficacy of neoadjuvant treatment has been found to be a function of the duration of the treatment. The majority of NET randomized trials use a treatment duration of 3 to 4 months, which is largely arbitrary and related to historical studies of tamoxifen and chemotherapy [17,18]. At the 2013 St. Gallen breast cancer conference, 62.2% of panelists supported NET being given until maximal response [19,20]. An additional 26.7% of panelists supported a duration of 4 to 8 months, while only 11.1% supported the current duration of 3 to 4 months [20]. One of the major concerns of extending NET until a maximal response is the risk of disease progression. A study by Carpenter et al., aiming to identify the optimal duration of letrozole therapy, had a low progression rate of 6.5% [17]. Similarly, a study of neoadjuvant exemestane showed a 7.7% progression rate at 4 months, increasing to only 8% at 6 months of treatment [21]. Despite the evidence, uptake of NET remained suboptimal, and it was stated that neoadjuvant ET in postmenopausal women with ER+ stage 2/3 tumors is currently underused, although it shows low toxicity when compared to neoadjuvant chemotherapy (panel discussion at the 2017 St. Gallen Conference) [22]. NET for up to 12 months is safe with close monitoring of tumor burden. It has also been noted that the following 12 months of treatment with different agents (and followed up to 13 years) presented no difference in disease-specific survival. The progression rate was significantly greater in the tamoxifen-only group at 80 months while compared with surgery followed by adjuvant tamoxifen [23].

**Figure 1:**
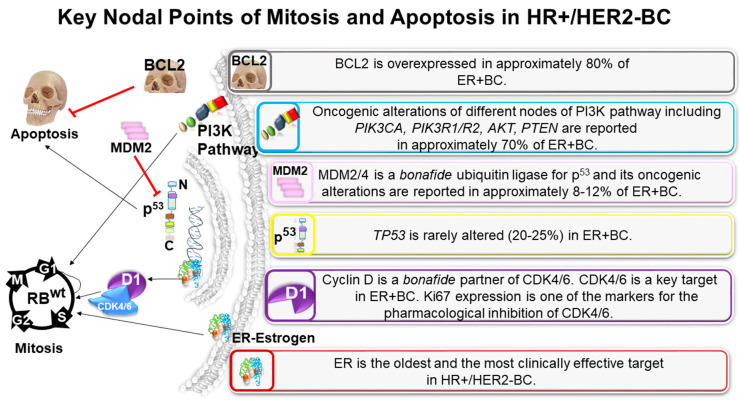
Key nodal points of mitosis and apoptosis in hormone receptor-positive (HR+)/human epidermal growth factor receptor 2-negative (HER2−) breast cancers (HR+/HER2−BC): major signaling pathways involved in proliferation and apoptosis in an HR+/HER2−BC tumor cell are presented. In a HR+/HER2−BC tumor cell, the major pro-proliferative signal involved in genomic and nongenomic functions of estrogen, which in the background of wild type TP53, transcriptionally regulates cell cycle via Cyclin D1. Cyclin D is a bona fide partner of CDK4/6. CDK4/6 is a key target in estrogen receptor-positive breast cancer (ER+BC). Cellular signals from the PI3K pathway are the primary survival pathway of the tumor cells, and activating (oncogenic/pathogenic) alterations of different nodes of the PI3K pathway, including PIK3CA, PIK3R1/R2, AKT, PTEN, are reported in approximately 70% of ER+BC. The most common antiapoptotic signal has been reported to be mediated via BCL2, and the protein is overexpressed in approximately 80% of ER+BC. Major signals in a HR+/HER2−BC tumor cell are presented in separate blocks. MDM4, a structural homolog of MDM2 that can form a heterocomplex with MDM2 and potentiate the ubiquitylation of p53. Please note that despite the conservation, the RING domain of MDMX has no detectable ubiquitin-ligase activity itself. MDM4 lacks an E3 ligase activity, but through heterodimerization, it regulates MDM2 enzymatic proficiency.

**Figure 2:**
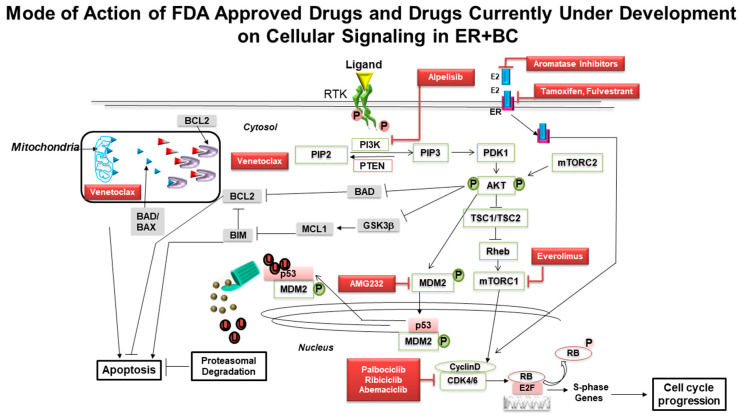
Mechanisms of actions of drugs, either FDA-approved or currently under development, on signaling nodes of Cyclin D1–CDK4/6–RB, PI3K/AKT/mTOR and apoptotic pathways in ER+ breast cancers: ER transcriptional activity and signaling through RTK/PI3K/AKT/mTOR increase Cyclin D levels thereby activating CDK4/6 and promoting cellular progression through the S phase by RB phosphorylation. Inhibition of AKT activates apoptosis via different cellular pathways, including P53-MDM2, BAD-BCL2, or GSK3beta-BIM-BCL2. Combined inhibition of different nodal points may help to develop new strategies for rationally designed novel clinical trials in the neoadjuvant setting with ER+ BC. The interactive signaling cascade in a tumor cell is complex. We tried to present it simplistically by including only the major/actionable/targetable/clinically relevant modal points in the context of ER+BC only. That this is why all other target genes of TP53 (PUMA, p21, NOXA) or ER-mediated AKT phosphorylation, or AKT-mediated ER phosphorylation, or nongenomic action of ER, SRK pathways are not included. Please note that the blue and red triangle inside the mitochondrial box represents BAD/BAX and venetoclax, respectively.

The outcome data of numerous trials using different modalities and timings of treatment(s) to block estrogen signaling to achieve clinical benefits has not been highly encouraging so far, inspiring scientists to search for further signaling avenues to perturb the estrogen pathway. The low pCR (pathologic complete response rate) obtained with tamoxifen or AIs alone does not make NET a suitable option for patients’ neoadjuvant treatment. Since a low pCR has been observed in HR+ BC following NET, there is now a focus on the role of biomarkers in disease progression and predicting treatment response, including de novo treatment resistance. The development of resistance has thrown a challenge both at the translational laboratories and current clinical practice to delve deep into the biology of the disease and its interaction with drugs (Figure 1). The mechanistic rationale now provides an opportunity to fish out different synergistic combinations whereby certain drugs’ clinical efficacy can be tested with the right biomarkers’ help favoring the outcome (Figure 2). Promising new treatments are being established and explored for the treatment of HR+ BC, including inhibitors of critical oncoproteins in the cell-cycle pathways (e.g., CDK4/6 inhibitors) and also in the PI3K-AKT-mTOR pathways, including isoform-specific PI3K inhibitor and mTORC1 inhibitor. A number of CDK4/6 inhibitors, including palbociclib, ribociclib, and abemaciclib, are now available and have demonstrated clinical efficacy in HR+ BC alongside antiestrogen therapy.

The purpose of the presentation of Figure 1 was to provide a simplistic representation of a very complex signaling cascade inside the tumor cell. The complex interactions purposely avoided giving a structured presentation of signaling key players responsible for mitosis and apoptosis in ER+ BC. This sets the groundwork for Figure 2, which presents a more complex signaling interaction of the ER and its cognate downstream effector molecules along with the PI3K-AKT-mTOR pathway. Since the signaling interaction is a complex interaction of inherently dynamic molecules and possesses both feedforward and feedback action in real time, we had to split the cartoon into a simple and complex presentation.

## 2. CDK4/6 Inhibitors and Endocrine Therapy

Several modes of inhibition of signaling initiated by estrogen-receptor have been tried in combination with chemotherapy/antiestrogen receptor therapy to achieve a better pCR for ER-positive BC. The premise for such treatment originated from the knowledge of downstream signals of the receptor activation in cancer cells empowered by the data from genomic alterations in the context of the cell-cycle. Cyclin D amplification/overexpression may be a promising biomarker for selecting a cell-cycle inhibitor along with ET. Cyclin D is the transcriptional target of ER, and its activation leads to an increased level of Cyclin D, which binds to CDK4/6, causing progression of the cell cycle.

One of the most recent successful treatment regimens used for HR+/HER2− metastatic BC is treatment with CDK4/6 inhibitors and endocrine therapy. A recent meta-analysis of data indicated that compared with ET alone, treatment with CDK4/6 inhibitors plus ET was associated with significantly improved OS, PFS, and objective response rate (ORR) among patients with ER+/HER2− metastatic BC [24]. In the same line, several studies have been initiated in the neoadjuvant setting with HR+ BC patients. The NeoPalAna trial, therefore, evaluated a CDK4/6 inhibitor in the neoadjuvant setting in 50 patients with ER+ early BC of both luminal A and B subtypes. Sequential biopsies were taken in patients who were initiated on anastrozole for 4 weeks, followed by the addition of palbociclib to study the additional change or decrease in Ki-67. The complete cell cycle arrest rate was significantly higher after adding palbociclib to anastrozole (87% vs. 26%, *p* < 0.001). The biomarker analysis suggested that response to palbociclib occurred independently of tumor grade, absence of progesterone receptor (PGR) expression, or mutation in *TP53*, *PIK3CA*, or *PTEN.* Resistance was associated with non-luminal subtypes and persistent E2F-target gene expression [25].

The clinical relationship between posttreatment reduction of Ki67 and the achievement of higher pCR has been evaluated to determine the therapeutic assessment of a treatment regimen. A neoadjuvant window-of-opportunity study (MONALEESA 1) with postmenopausal early-stage HR+/HER2− BC patients were randomized 1:1:1 to receive 2.5 mg/day letrozole alone (Arm 1) or with 400 or 600 mg/day ribociclib (Arm 2 or 3). Circulating tumor DNA (ctDNA) and tumor biopsies were collected at baseline and, following 14 days of treatment, prior to or during surgery. The results showed that Ki67 levels decreased in the two combination arms compared to letrozole alone (69% decrease in the letrozole arm, and over 90% decrease in both Arm 2 and 3) [26].

A PALLET trial was initiated in ER+/HER2− patients in the neoadjuvant setting with letrozole plus palbociclib. Three hundred seven patients were recruited. Clinical response was not significantly different between palbociclib plus letrozole vs. letrozole groups (*p* = 0.20; complete response + partial response, 54.3% vs. 49.5%), and progressive disease was 3.2% versus 5.4%, respectively. Adding palbociclib to letrozole significantly enhanced the suppression of malignant cell proliferation (expression of Ki-67) over 14 weeks [27]. Similarly, a phase 2 neoMONARCH study in 224 HR+/HER2− early-stage BC patients with abemaciclib and anastrozole demonstrated a significant decrease in Ki67 expression and led to the potent cell-cycle arrest. More patients in the abemaciclib-containing arms versus anastrozole alone achieved complete cell-cycle arrest (58%/68% vs. 14%, *p* < 0.001). Importantly, the combination therapy maintained inhibition of cell proliferation and led to the activation of the T-cell immune response. Genetic data revealed that the presence of a *PIK3CA* mutation had no significant effect on Ki67 expression change from baseline to 2 weeks, including the rate of complete cell cycle arrest in response to abemaciclib alone (*p* = 0.57) or in combination compared to anastrozole. Resistant tumors displayed a numerically higher expression of *CCNE1* and *RB* loss-of-function score than sensitive tumors, although this was not statistically significant [28]. Although a reduction of Ki67 has been reported in most cases, this reduction of Ki67 has not been found to be associated with the improvement of pCR.

From these above-mentioned studies, it is not clear whether the combination of CDK4/6 inhibitors plus ET is more effective than chemotherapy in a neoadjuvant setting with HR+/HER2− BC patients. The NeoPAL study evaluated palbociclib plus letrozole with chemotherapy in high-risk luminal BC patients. The primary endpoint was the residual cancer burden (RCB). Secondary endpoints included clinical response, proliferation-based markers, and safety. A total of 106 patients were randomized. RCB was observed in 7.7% in the palbociclib plus letrozole arm and 15.7% in the chemotherapy arm. Pathological complete response rates were 3.8% and 5.9%. Clinical response (75%) and breast-conserving surgery rates (69%) were similar in both arms. As expected, the safety profile was worse in chemotherapy with 2 versus 17 serious adverse events (including 11 grade 4 serious AEs in the chemotherapy arm) [29]. Recently, treatment data (with ribociclib plus letrozole versus chemotherapy) from 106 postmenopausal women with stage I-IIIA, HR+/HER2−, luminal B (by PAM50) tumors were found to be not specifically encouraging (published CORALLEEN phase 2 trial). Data suggested that some patients with high-risk, early-stage, HR+/HER2− BC could achieve molecular downstaging of their disease with ribociclib and letrozole [30].

## 3. PI3K Pathway Inhibitors and Endocrine Therapy

The PI3K-AKT-mTOR pathway is one of the pathways associated with the activation of ER+ BC, and its activation is known to be responsible for the failure of antiestrogen therapy. The PI3K-AKT-mTOR pathway modulates responses to signals communicated through the ER and HER family of receptors in BC. The pathway is critical to determine the clinical sensitivity of BC to endocrine therapy. Currently, everolimus and alpelisib have been approved by the FDA for the treatment of ER+ BC. BOLERO-2 trial also established the clinical synergism between AI and everolimus in ER+ advanced BC patients [31]. Before this study, Baselga’s group revealed that the clinical response rate was significantly higher in a combination of everolimus plus letrozole compared to letrozole alone in the neoadjuvant setting ER+/HER2− BC patients [32]. It was demonstrated from the analyses of the BOLERO-2 trial that a greater benefit from everolimus treatment was obtained in patients with minimal genetic alterations in *PIK3CA/PTEN/CCND1* or *FGFR1/2* genes. When compared, patients with a single alteration in one of these pathways exhibited a median progression-free survival of 214 days with everolimus as compared to 77 days with placebo (hazard ratio [HR] = 0.26 (ASCO Post 2013). In contrast, alpelisib has been recommended in the situation of activating mutation of *PIK3CA* in the tumor.

Based on the mutational repertoire, the *PIK3CA* mutation is the most prevalent gain-of-function mutation (~40%) in ER+ luminal BC. The alpha catalytic isoform selectively and rightfully makes a powerful argument for highly specific *PIK3CA* targeting drug candidates [33]. Recently, alpelisib (an alpha isoform-specific PI3K inhibitor) has been FDA approved following the successful SOLAR-1 phase III trial in ER+ advanced BC [34]. In contrast to the results of the SOLAR 1 study in advanced/metastatic disease, the addition of alpelisib to 24-week neoadjuvant letrozole treatment did not improve response in patients with HR+ early BC. The pCR rates were low in letrozole plus alpelisib and placebo groups. Decreases in Ki-67 were similar across treatment arms and cohorts. In *PIK3CA*-mutant tumors, alpelisib plus letrozole treatment induced a greater target engagement in terms of a decrease in phosphorylated-AKT versus placebo plus letrozole [35]. The phase-2 LORELEI trial is also not an exception. This trial evaluated the efficacy of letrozole plus taselisib (beta- sparing PI3K inhibitor) or placebo in patients with operable HR+/HER2− BC patients with stage 1–3. The addition of taselisib to letrozole was associated with a higher proportion of patients achieving an objective response in all randomly assigned patients (39% patients in the placebo group vs. 50% in the taselisib group; *p* = 0.049) and in the *PIK3CA* mutant subset (38% vs. 56%; *p* = 0.033). No significant differences were observed in pCR between the two groups either in the overall population (2% in the taselisib group vs. 1% in the placebo group; OR 3.07 (*p* = 0.37) or in the *PIK3CA* mutant cohort (1% vs. 0%; OR not estimable, *p* = 0.48) [36].

The TRINITI-1 trial with advanced HR+/HER2− BC with a triplet-therapy (ribociclib plus everolimus plus exemestane) demonstrated clinical benefit at week 24 in 41.1% patients, exceeding the predefined primary endpoint threshold (>10%) [37]. The presence of *PIK3CA* mutations in liquid biopsy correlated with shorter PFS (7.44 vs. 12.9 months) in patients receiving CDK4/6 inhibitor plus hormone therapy treatment [38]. Taken together, it is worth perusing clinical trials with triple combinations (CDK4/6 inhibitors plus alpelisib or everolimus plus antiestrogen) in a neoadjuvant setting to improve pCR with HR+/HER2−/*PIK3CA*-mutated BC patients.

The power to identify different biomarkers of a drug and the ability to chart its role in cell signaling has empowered us to predict and propose the subsequent development of resistance to a drug or a combination. PI3K pathway hyperactivation due to *PIK3CA* mutations contributes to endocrine resistance. Several routes to the development of resistance have been observed involving additional oncogenic pathways, de novo, or acquired drug treatment. Thus an effective drug can be rendered ineffective. Cyclin-dependent kinase 4 and 6 inhibitors (CDK4/6i) have changed the HR+/HER2− BC treatment landscape. Putative mechanisms of resistance to CDK4/6 inhibitors have been identified, but limited data are available on PI3K deregulation [38]. Dysregulation of the PI3K pathway, including *PIK3CA*, *PTEN,* and *AKT* kinase, leads to an increase in cell proliferation and survival following the disruption of apoptosis. As expected, alpelisib, and everolimus have been routinely used in clinics.

The data from recent studies are promising, even though they are less impressive than expected when compared to the metastatic setting. In this context, the use of genomic/transcriptomic approaches (e.g., ONCOTYPE, PAM50) and the identification of novel biomarkers (ESR1, PI3KCA, BCL2) on tissue or with liquid biopsy could help to select patient prone to respond to endocrine-combined therapy and able to achieve high pCR.

## 4. Traveling Forward

The biology of the disease is the single most critical determinant for the (1) choice of targeted therapy and response of the tumor cells to the drugs; (2) outcome of the disease; (3) development of resistance, de novo or therapy-induced; and (4) the metastatic progression of the disease. However, a combination of ET plus targeted therapy in the neoadjuvant setting decreased Ki67 staining (as an indicator of low proliferation), could not yet achieve the desired pCR. Since evading of apoptosis is a classical hallmark of cancer, and venetoclax (a BCL2 inhibitor) has shown clinical benefit in combination with tamoxifen, we argue that drugs inducing apoptosis should be considered to be included in the treatment regimen. Against the backdrop of the genomic alterations in the ER+/HER2− BC, the cell signaling associated with these alterations, and different therapeutic combinations tested in various trials, we propose two scenarios to induce apoptosis. The proposed combinations are contextual and based on the current understanding of the cell signaling in ER+/HER2− BC.

## 5. A Perfect Apoptosis Plot in HR+/HER2−BC

Tumor growth is associated with the loss of balance between the rate of mitosis and apoptosis. In a tumor cell, the proliferative signals are by default integrated to the apoptotic signals in such a way that a tumor cell committed to increased proliferation cannot have increased apoptosis at the same time. The uncertainty arises when this default signaling is disrupted in the face of two events, (1) oncogenic transformation and (2) the first line of therapy following clinical identification of the disease in patients. In specific cancer cells, paclitaxel, or other spindle poisons upregulate antiapoptotic BCL2 family members and/or decrease the expression of proapoptotic BAX [39]. Expression of Bcl-xL and the loss of *TP53* have been reported to cooperate to overcome a cell cycle checkpoint induced by mitotic spindle damage [40].

## 6. Priming Apoptosis: Cell-Cycle Inhibitor Plus BCL2 Inhibitor

**Figure 3:**
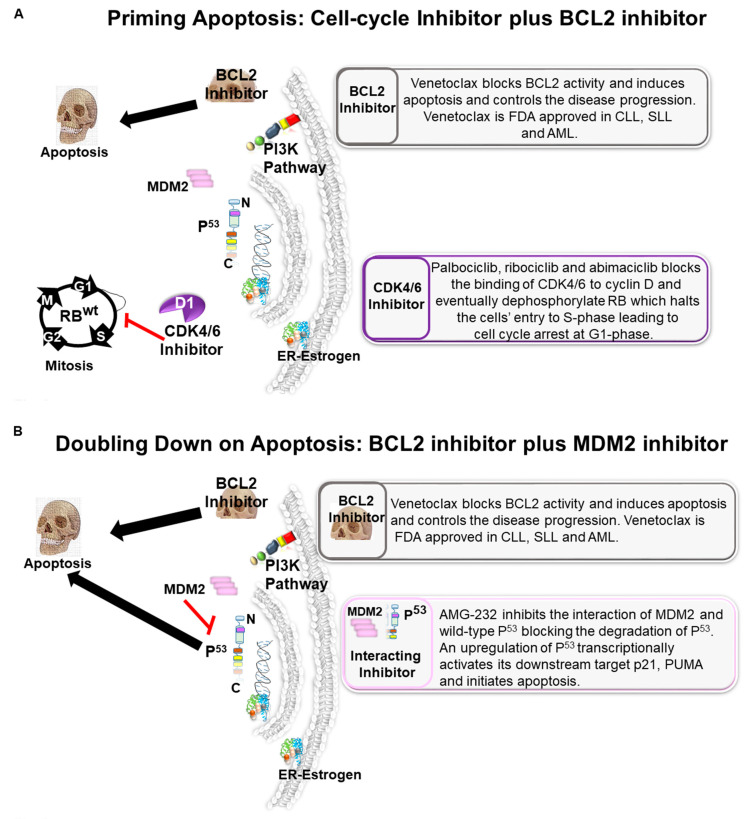
A plot diagram to prime for apoptosis (**A**) and doubling down on apoptosis (**B**) using inhibitors of BCL2, cell cycle, and MDM2 in ER+BC: the schematic representation of a proposed mode of action of induction of apoptosis by the inhibition of BCL2 in combination with (**A**) inhibition of CDK4/6, and (**B**) inhibition of MDM2 in an HR+/HER2−BC tumor cell are presented in separate blocks. The figure is a pictorial representation of the two concepts that we have proposed in ER+BC to induce apoptosis. They are (1) to prime for apoptosis using BCL2 inhibitor plus CDK4/6 inhibitor and (2) doubling down on apoptosis using inhibitors of BCL2 and MDM2 in ER+BC in the neoadjuvant setting.

BCL2 protein blunts activation of the mitochondrial pathway to apoptosis, and it is overexpressed in approximately 80% of ER+ BC. Venetoclax blocks BCL2 activity and induces apoptosis, and controls disease progression [41,42]. Cyclin D is the transcriptional target of estrogen, and it is a crucial target in ER+ BC. The Ki67 expression monitors pharmacological inhibition of Cyclin D. Palbociclib, ribociclib, and abemaciclib block the binding of CDK4/6 to Cyclin D and eventually dephosphorylates RB, which halts the cells’ entry to S-phase, leading to cell cycle arrest at G1-phase. Apoptosis of a tumor cell which is locked in the G1 (static) phase can be additively primed by concurrent inhibition of BCL2 (Figure 3A).

A couple of years back, Lok et al. presented a new concept to treat ER+ metastatic BC patients using venetoclax (a potent and selective BCL2 inhibitor that has shown increased apoptotic response and achieved FDA approval in the CLL (chronic lymphocytic leukemia), SLL, (small lymphocytic lymphoma), and AML (acute myeloid leukemia) settings) [41]. BCL2-BAD/BAX is one of the key pathways to evade cell death. Studies published by Prof. G. J. Lindeman’s team demonstrated the efficacy of a combination of venetoclax plus endocrine therapy, confirming that the radiological response rate was 50% and clinical benefit rate was 75% in ER+ BCL2+ metastatic patients with BC [41]. Their findings support further investigation of combination therapy for patients with ER+/BCL2+ BC. In 2000, Perillo et al. [43] showed that BCL2 expression could be upregulated as a downstream effector molecule during ER stimulation, and it has been reported that approximately 85% of primary ER+ BC demonstrate BCL2 overexpression [44]. The demographic predominance of BCL2 overexpression represents another promising therapeutic target in ER+ BC, along with NET. Since the addition of CDK4/6 inhibitor to AI markedly enhanced the suppression of malignant cell proliferation as measured by Ki-67 expression, yet did not achieve high pCR (<5%), it is possibly related to a lack of concurrent high apoptosis. Whittle and colleagues recently reported a preclinical proof-of-concept study wherein they demonstrated an augmented tumor response in ER-positive BC by treating ER+ BC cell lines (also PDX) with fulvestrant plus palbociclib and venetoclax [45]. This study supports the investigation for targeting BCL2 in combination with CDK4/6 inhibitor and an antiestrogen as targeted neoadjuvant therapy in ER+ BC.

## 7. Doubling Down on Apoptosis: BCL2 Inhibitor Plus MDM2 Inhibitor

Cell-cycle arrest and apoptosis are the most superior outcomes of the transcriptional activation of *TP53*, which are critical for cell fate decision and, hence, prevention of tumor development. The discovery that p53 can act negatively to block transformation and can act as a suppressor of transformation by Levine and colleagues [46] paved the path for extensive studies in the future. Since p53 functions as a physiological rheostat connecting cell proliferation and apoptosis safeguarding against damaged, potentially malignant cells, several oncogenic signals have been reported to offset this signaling in the course of transformation. One such event is reported to be via alteration of the apoptotic threshold by direct transcription activation or repression of BCL2 family proteins [47]. Thus gaining the ability to fine-tune p53 ability to determine cell fate by apoptosis can have meaningful therapeutic potential. Furthermore, p53 has been reported to have a complex cross-talk with the pRb pathway through MDM2-pRb binding [48]. As an evolutionarily conserved function, p53 induces an array of genes involved in apoptosis [49], including BH3 domain-only proapoptotic proteins, death receptors and apoptosis execution factors. The function of p53 is extended to the mitochondria beyond transcription function in the nucleus, and the most important function in the context of tumorigenesis involves BCL2 function. BH3-only proteins induced by p53 causes mitochondrial outer membrane permeabilization (MOMP), a critical step in the intrinsic apoptosis pathway [50]. MOMP is required for the activation of execution caspases and is a readout of apoptosis in several cancer cells following anticancer drugs [51]. Hence, mitochondrial outer membrane integrity is regulated primarily through interactions between proand antiapoptotic members of the BCL-2 protein family, a readout of the balance of proand antiapoptotic BCL2 family proteins. p53 binds to BCL2 and BCLxL [52,53], releasing and activating BAX and BAK. The p53 core domain directly interacts with proapoptotic BAK, relieving BAK from inhibitory complexes with MCL1 [54,55]. One way to reinstate p53 functions in the clinical setting was to stabilize p53 function, posttranslationally, by blocking the MDM2 action.

In contrast to *BCL2*, *TP53* is rarely altered (20–25% genomic alteration) in ER+ BC. MDM2 is a bona fide ubiquitin ligase for p53 protein, and its oncogenic alterations are reported in approximately 8–12% of ER+ BC. AMG-232 inhibits the interaction of MDM2 and wild-type p53, blocking the degradation of p53. We propose that inhibition of BCL2 function in the background of enhanced p53 signaling of wild type *TP53* following inhibition of MDM2 will double-down on the status of apoptotic signals in tumor cells (Figure 3B). Since both BCL2 and MDM2 are overexpressed in BC and *TP53* is rarely mutated in the ER+ disease, testing a dual inhibition of BCL2 and MDM2 signals warrants further investigation.

Although in the neoadjuvant treatment landscape, many ongoing studies are aiming to evaluate the association of new target molecules with ET, the observed overall low pCR does provoke thoughts to consider the different therapeutic approaches. One logical approach is to induce apoptosis by targeting BCL2 along with the p53-MDM2 axis. The recently surfaced interest in TP53-MDM2 axis inhibition is gaining traction clinically. Three trials are currently studying a potent and selective MDM2 inhibitor, AMG-232, in various cancer settings. AMG-232 appears to rely on WT *TP53*; it does not affect mutant *TP53*. AMG-232 binds to MDM2, prevents the ubiquitination of WT p53 by its negative regulator MDM2 and allows p53 to fulfill its important proapoptotic role. Approximately 8–12% of ER+ BC are *MDM2*-amplified/overexpressed, and 75% were *TP53* WT within the Cancer Genome Atlas (TCGA) cohort and cohorts from the Avera Cancer Center [56]. A preclinical in vivo study with endocrine-sensitive and resistant breast cancer cell lines by Lu et al., using another MDM2 inhibitor, MI-77301, showed significant efficacy without any evidence of toxicity in mice [57]. From these studies mentioned above, it is logical to organize a novel trail design utilizing triple therapy with BCL2 inhibitor, p53-MDM2 interacting inhibitor, and an antiestrogen. The patient population’s molecular stratification based on the *TP53* status (WT), BCL2 overexpression, and *MDM2* amplification will give a better chance of predicting outcome.

## 8. Conclusions

Targeted therapy is a promising direction but likely necessitates the identification of predictive/actionable biomarker(s) to guide the therapy. In the era of precision medicine, with the development of NGS, tumor-biopsy and blood-based biopsy are employed to understand tumor biology and biomarker(s) and hence identify novel targets. The data help to elucidate the development of resistance, as well as improve neoadjuvant targeted therapies to the next level, i.e., biomarker-based patient-specific combination therapies or n-of-1 like trial designs.
